# Pneumonia with Hyperpyrexia

**Published:** 1898-03

**Authors:** E. V. Ross

**Affiliations:** 279 Jefferson Avenue; Rochester, N. Y.


### PNEUMONIA WITH HYPERPYREXIA.

E. V. Ross, M. D., Rochester, N. Y.

In the December number of The Homœopathic Physician, under the heading, "Some Indications Derived from the Bed," among other things we read: "Think some one is in the bed with him, Apis, Petroleum. It is characteristic of Petroleum."

This prompts us to make the following report of a recent case wherein this symptom was prominent, and led to a brilliant cure:

Charles H., aged twenty, medium height, well nourished, was taken on January 1st with a prolonged chill. I saw him the following day at 7 P. M., and found his symptoms to be as follows: Cough, with frothy, blood-streaked mucus; thick, milky-white coating on tongue, having a yellowish tinge; loss of appetite; great thirst, drinks often and about one-half glass at a time; vomits after drinking; lame, sore feeling all over; great prostration; dark red flush over malar bones; feeling as if another chill would come on if he moved; no movement from bowels for past two days; urine scanty; no crepitation or dullness; vocal fremitus but slightly increased; temperature, 106 F.; pulse, 120, weak, compressible; respiration, 30. R. Bryonia, 2m (Jenichen), two powders to be given one hour apart, then S. L. Diet, all the milk he will take.

January 3d, 10 A. M.—Is now very delirious, wandering delirium; talks of various things. Restlessness, says the bed is hard; vomiting soon after drinking; refuses all nourishment, but craves beer; cough worse; sputa "rusty" colored and shiny; sweats by spells on nose and face, but soon dries off; temperature, 106.2; pulse, 140, weak, compressible; respiration, 36. 10 P. M.—Tongue has a dry, cracked appearance across dorsum; lips dry; eyes have a wild, staring look; face yellow, with a central flush; no rales, no typanitic dullness; urine scanty; highly-colored s. g. 1030; albumen marked; temperature, 106.6; pulse, 142; dicrotism marked, respiration, 38; otherwise same as in A. M. R. Sulphur, 4500 (Jenichen). One powder. S. L. to follow.

January 4, 10 A. M.—Very delirious during night; tried to get out of bed; now and then thinks there is some one in bed with him; sordes on lips; tongue very dry and crocked; sputa abundant, "rusty," and occasionally it is profusely streaked with blood of a "prune-juice" color; temperature, 106.8; pulse, 144; respiration, 40. 11 P. M.—All symptoms worse; does not recognize any one; the delusion that some one is in the bed with him is now quite constant; vomiting better; temperature, 107; pulse, 148, very weak, and compressible, respiration 44; tactile fremitus increased; absence of crepitation and dullness; no stool to date; urine scanty, dark. R. Baptisia 20m (fluxion), in solution. Teaspoonful hourly until better. January 5, 11 A. M.—Was sleeping quietly when I called, and in a drenching perspiration. Relatives informed me that he "quieted down" after midnight, and about 2 A. M. fell asleep. When he awoke he appeared to be quite rational, and greeted me with, "Good-morning, doctor!" and only now and then did he appear to be slightly muddled. Temperature, 100.5 F.; pulse, 80, stronger; respiration, 24; tongue moist and sordes peeling off lips. In brief, he rapidly convalesced. Three days later he sat up in a chair, and had nothing to complain of except a craving appetite.

Remarks: Wunderlich applies the term hyperpyrexia to temperatures which approach and exceed 107.6 F. According to this statement, our case can be considered as one having an excessively high temperature. The vast majority of pneumonias with a rising temperature approaching the 107 mark terminate fatally.

The temperature was ascertained by two certified thermometers of different make—Hicks and Weinhagens—and both tallied.

Another feature of the case was the absence of any physical signs,* except the increased tactile fremitus discernible on the fourth day, the pathologist would call this a case of central pneumonia, as the process of hepitization was evidently working from centre to periphery when Baptisia suddenly interrupted its onward course and brought about the crisis.

Pneumonia now and then fails to pursue the typical course that is laid down in the text-books, and we occasionally see atypical cases, wherein one or more of the classical symptoms, as chill, cough, rapid respiration, pain in side, disturbance in the pulse-respiration ratio (Normal 2-9 Juergensen), dullness, crepitation, "rusty" sputa are absent. Especially is this true of pneumonia occurring in people past middle life, and the so-called secondary pneumonia, and in spite of the fact that it is the cause of death in fully one-half of the cases occurring in those of sixty years and over. It is frequently overlooked, and this is so for the very reason that the diagnostic symptoms are lacking in many cases.

The guiding symptom that led to the selection of Baptisia was the delusion that some one was in bed with him. The only remedy found in the materia medica as having this exact symptom to its credit is *Petroleum*, but it did not cover the other symptoms. Under *Baptisia* the patient imagines that he is double; that his body is scattered about the bed, etc. This could be construed as an analagous mental state. Lee's *Repertory of the Mind and Head* assisted us materially, for the following is therein recorded (p. 27): "Feels as if some one was in bed with him, Anac., Apis., *Bapt.*, Carbo-v., Nux-v., Op., Petrol., *Puls.*, Rhus-t., Valer."

---

*Owing to the marked delirium and the high temperature, we looked well to the apical region, but failed to elicit any dullness or crepitation.

This, coupled with sore feeling of the body, offensive breath, sordes on lips, the yellow appearance of face, with a central flush over malar bones, decided the choice in favor of Bapt., and its action was prompt and decided.

279 Jefferson Avenue.

